# Classification of Benign Hematogones and B-cell Acute Lymphoblastic Leukemia in Peripheral Blood Smear Images Using Artificial Intelligence

**DOI:** 10.7759/cureus.114985

**Published:** 2026-08-22

**Authors:** Mark A Bachir, Neel Nawathey, Alex Bachir, Akshay J Reddy, Simran Gill, Rakesh Patel

**Affiliations:** 1 Internal Medicine, California Northstate University College of Medicine, Elk Grove, USA; 2 Osteopathic Medicine, Touro University California, Mare Island, USA; 3 Medicine, California Health Sciences University, Clovis, USA; 4 Medicine, California University of Science and Medicine, Colton, USA; 5 Medicine, University of California, Los Angeles, Los Angeles, USA; 6 Internal Medicine, East Tennessee State University Quillen College of Medicine, Johnson City, USA

**Keywords:** artificial intelligence, automated image analysis, b-cell acute lymphoblastic leukemia, benign hematogones, deep learning, flow cytometry, hematopathology, image classification, medical imaging, peripheral blood smear

## Abstract

B-cell acute lymphoblastic leukemia (ALL) is a hematologic malignancy characterized by the abnormal proliferation of immature B-lineage lymphoblasts. Automated analysis of peripheral blood smear images may improve the speed and consistency of cell classification while supporting specialist interpretation. In this study, a cloud-based artificial intelligence image classification model was developed to differentiate benign hematogones from early pre-B, pre-B, and pro-B ALL categories. The model was trained and evaluated using 3,208 microscopic images from a publicly available dataset. Images were divided into training, validation, and testing subsets containing 2,568, 320, and 320 images, respectively, and model development was performed using Google Cloud AutoML (Google LLC, California, US). Performance was evaluated using average precision, precision, recall, precision-recall analysis, confidence-threshold evaluation, and a multiclass confusion matrix. At a confidence threshold of 0.50, the model achieved an average precision of 1.00, a precision of 100%, and recall of 99.4%. Class-specific correct classification rates were 100% for benign hematogones, early pre-B ALL, and pro-B ALL, and 98% for pre-B ALL, with the remaining pre-B images classified as early pre-B ALL. These findings demonstrate strong image-level performance within the internal testing dataset. External, multi-institutional, and patient-level validation is required before the model can be considered for clinical application.

## Introduction

Acute lymphoblastic leukemia (ALL) is an aggressive hematologic malignancy characterized by the uncontrolled proliferation of immature lymphoid cells in the bone marrow, peripheral blood, and other tissues. In the United States, ALL has an age-adjusted incidence of approximately 1.9 cases per 100,000 persons per year, with an estimated 6,250 new cases in 2026, and it is most frequently diagnosed in individuals younger than 20 years. The accumulation of lymphoblasts disrupts normal hematopoiesis and may lead to anemia, thrombocytopenia, infection, bleeding, and extramedullary disease. Although ALL occurs in both children and adults, it is the most common leukemia diagnosed in childhood. B-lineage ALL accounts for most ALL cases and comprises biologically diverse diseases with important differences in genetic alterations, treatment response, and prognosis [1,2].

The classification of B-ALL has evolved alongside advances in molecular and genetic characterization. Current World Health Organization and International Consensus Classification systems define many B-ALL entities according to recurrent genetic abnormalities in addition to morphology and immunophenotype [3,4]. Immunophenotypic classification is primarily determined by flow cytometry using patterns of B-lineage and maturation marker expression; pro-B cells represent the least differentiated phenotype, early pre-B cells typically express markers such as CD10 without cytoplasmic immunoglobulin μ heavy chain, and pre-B cells demonstrate cytoplasmic μ heavy-chain expression while lacking surface immunoglobulin [3-5]. Terms such as pro-B, early pre-B, and pre-B describe immunophenotypic patterns associated with stages of B-cell development. These categories are useful for characterizing cellular phenotype but should not be interpreted as sequential clinical stages through which leukemia necessarily progresses [3-5].

The diagnosis of suspected ALL requires integration of clinical findings with laboratory evaluation. Complete blood counts and microscopic examination of peripheral blood or bone marrow smears may provide an initial assessment by identifying abnormal lymphoblasts. However, morphology alone is generally insufficient to establish lineage or determine the complete disease subtype. Flow cytometry is used to evaluate lineage-associated and immaturity markers, while cytogenetic and molecular studies identify abnormalities that influence classification, prognosis, and treatment selection [5,6]. Standardized flow cytometry panels have further improved the reproducibility of immunophenotypic analysis and the distinction between normal, reactive, and malignant cell populations [7,8].

A particularly important diagnostic challenge is differentiating B-ALL lymphoblasts from hematogones. Hematogones are benign B-cell precursors that may be present in peripheral blood or bone marrow and can share morphologic and immunophenotypic characteristics with malignant lymphoblasts. Multiparameter flow cytometry can usually distinguish these populations by evaluating coordinated patterns of antigen expression, but accurate interpretation depends on appropriate laboratory methods and specialist expertise [9,10]. This distinction is central to the present study because tarhe benign category in the analyzed dataset represents hematogones rather than mature normal leukocytes.

Microscopic examination remains valuable because it is rapid, widely available, and provides direct information about cellular morphology. Nevertheless, manual interpretation can be labor-intensive and may be influenced by staining quality, illumination, magnification, image resolution, and observer experience. Artificial intelligence (AI) may support this process by detecting visual patterns within microscopic images. Deep-learning models, particularly convolutional neural networks, can learn features such as cellular shape, nuclear appearance, texture, color, and spatial organization directly from image data and have increasingly been applied to hematologic image analysis [11].

Previous studies have demonstrated that AI can distinguish ALL cells from benign cells and classify leukemia subtypes with high internal performance [12-18]. These investigations have generally reported strong internal performance, suggesting that automated models can recognize visual differences among benign B-cell precursors and leukemic lymphoblasts. However, performance may be influenced by dataset size, class distribution, image preprocessing, training strategy, and evaluation design. High accuracy within an internally divided public dataset does not necessarily indicate that a model will perform similarly across different hospitals, microscopes, staining protocols, or patient populations. Transparent reporting of dataset composition, partitioning, model development, performance metrics, and study limitations is therefore essential when evaluating AI systems in medical imaging [19].

Despite promising findings from prior studies, the performance of accessible cloud-based platforms for distinguishing benign hematogones from related B-ALL categories remains insufficiently characterized. In the present study, we developed a Google Cloud AutoML (Google LLC, California, US) image-classification model to distinguish among four categories of peripheral blood smear images: benign hematogones, early pre-B ALL, pre-B ALL, and pro-B ALL. Images were obtained from the publicly available ALL Image Dataset, which contains specialist-labeled microscopic images classified using flow cytometry as the reference method [20]. The objective of this study was to determine whether an automated cloud-based platform could differentiate benign B-cell precursors from B-ALL lymphoblasts while also distinguishing among the three B-ALL categories represented in the dataset. The model was evaluated as an image-level classification system and was not intended to replace flow cytometry, molecular testing, or expert hematopathologic interpretation.

## Materials and methods

Dataset and study design

Microscopic peripheral blood smear images were obtained from the publicly available ALL Image Dataset hosted on Kaggle [20]. The final analysis included 3,208 images assigned to one of four categories: benign hematogones, early pre-B ALL, pre-B ALL, or pro-B ALL. The category labels provided with the source dataset were used as the reference labels for model development and evaluation.

Images were divided into training, validation, and testing subsets using an 80:10:10 distribution. The training subset contained 2,568 images, while the validation and testing subsets each contained 320 images. The training subset was used for model development, the validation subset was used by the automated platform during model optimization, and the testing subset was reserved for final performance evaluation.

The publicly available dataset did not provide sufficient patient-level identifiers to confirm that all images originating from the same patient were retained within a single subset. Therefore, the analysis should be considered an image-level evaluation, and patient-level separation among the training, validation, and testing subsets could not be verified.

Model development

The image-classification model was developed using Google Cloud AutoML. A single-label, multiclass configuration was selected so that each blood smear image could be assigned to one of the four possible categories.

Feature extraction, model optimization, and hyperparameter selection were performed automatically by the platform. Internal model specifications, including the neural-network architecture, learning rate, batch size, optimization method, and other training parameters, were not manually selected or made available to the investigators. Consequently, these platform-controlled settings could not be independently reproduced or modified.

For each image in the testing subset, the model generated a probability score for all four categories. The category with the highest predicted probability was recorded as the final classification. A confidence threshold of 0.50 was selected for the primary evaluation of model performance.

Performance evaluation

Model performance was evaluated using average precision, precision, recall, precision-recall analysis, confidence-threshold analysis, and a multiclass confusion matrix. Performance values were obtained from the evaluation outputs generated by Google Cloud AutoML. Average precision, precision, and recall range from zero to one, with values closer to one indicating better performance.

Average precision summarizes performance across confidence thresholds by integrating the relationship between precision and recall. Precision represents the proportion of positive model predictions that are correct and is useful for assessing false-positive predictions, whereas recall represents the proportion of true instances that are correctly identified and is sensitive to false-negative predictions. Because increasing precision may reduce recall and vice versa, neither metric should be interpreted in isolation.

The precision-recall curve illustrates this tradeoff across different confidence thresholds, with curves approaching the upper-right region indicating stronger performance. Confidence-threshold analysis was used to evaluate how changing the minimum probability required for classification affected precision and recall. A threshold of 0.50 was used for the primary analysis.

The multiclass confusion matrix compared the reference category of each testing image with the category predicted by the model. Correct classifications appeared along the diagonal, while off-diagonal values represented misclassifications between categories. Unlike precision and recall, the confusion matrix does not provide a single numerical performance score but allows direct visualization of class-specific errors and the categories most frequently confused by the model. Performance was examined separately for benign hematogones, early pre-B ALL, pre-B ALL, and pro-B ALL.

Representative images from each category were reviewed to illustrate the model’s predicted classification and corresponding probability scores. These examples were presented only to demonstrate model output and were not used as substitutes for evaluation of the complete testing dataset.

Ethical considerations

Institutional review board approval was not required because the study used only publicly available microscopic images. No participants were directly enrolled, and no protected health information was accessed.

## Results

Overall model performance

The final model was evaluated using 320 peripheral blood smear images from the testing subset. At the selected confidence threshold of 0.50, the model achieved an average precision of 1.00, a precision of 100%, and recall of 99.4%. These findings demonstrate strong overall classification performance within the internal testing dataset.

**Figure 1:**
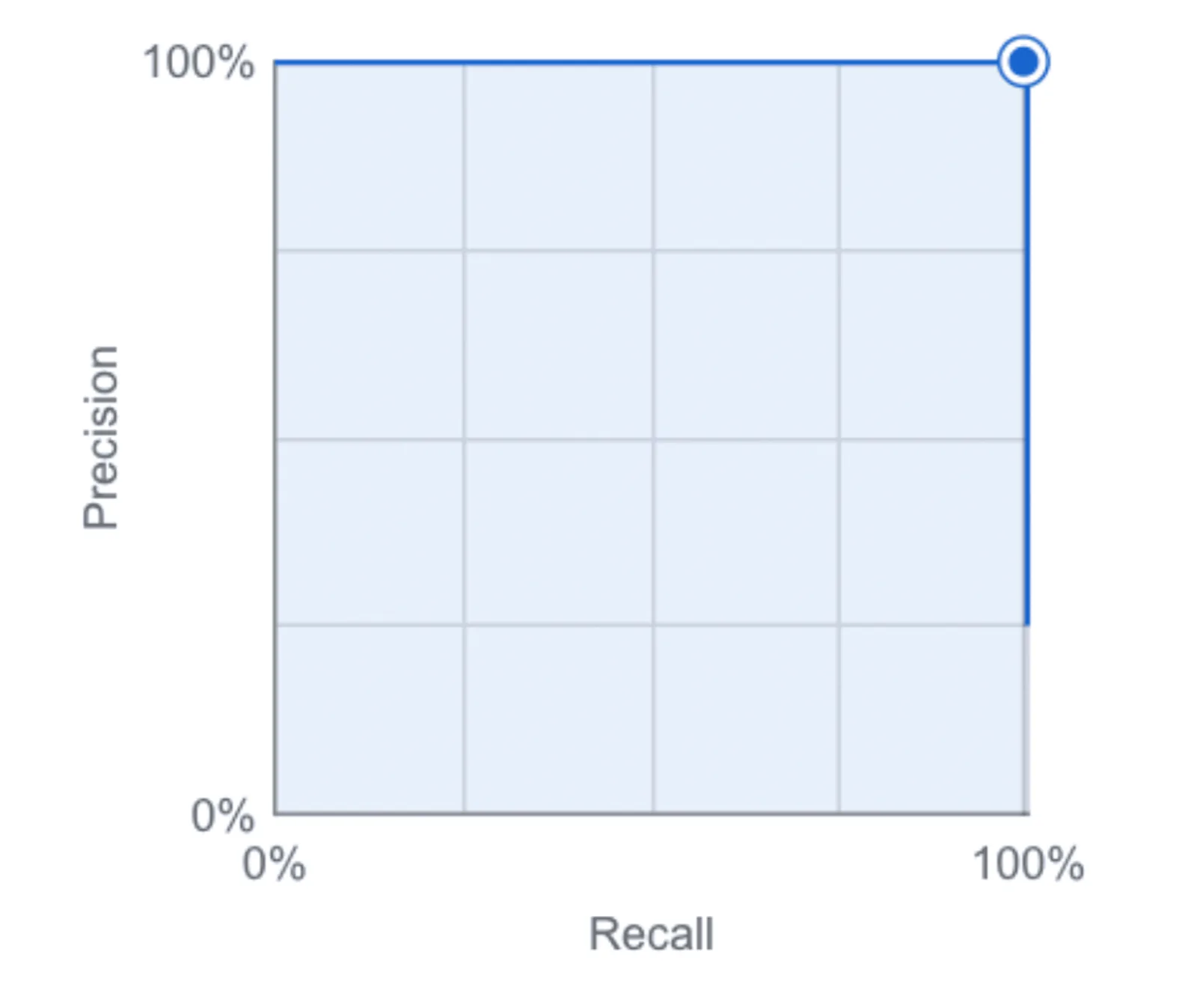
Precision-recall performance of the blood smear classification model The precision-recall curve shows the relationship between model precision and recall across the evaluated operating range. Precision remained near 100% through most of the curve, indicating consistently strong classification performance.

The precision-recall curve showed consistently high performance across the evaluated operating range. Precision remained near 100% through most of the curve, while recall remained high across a broad range of classification thresholds (Figure 1).

Confidence-threshold analysis

**Figure 2:**
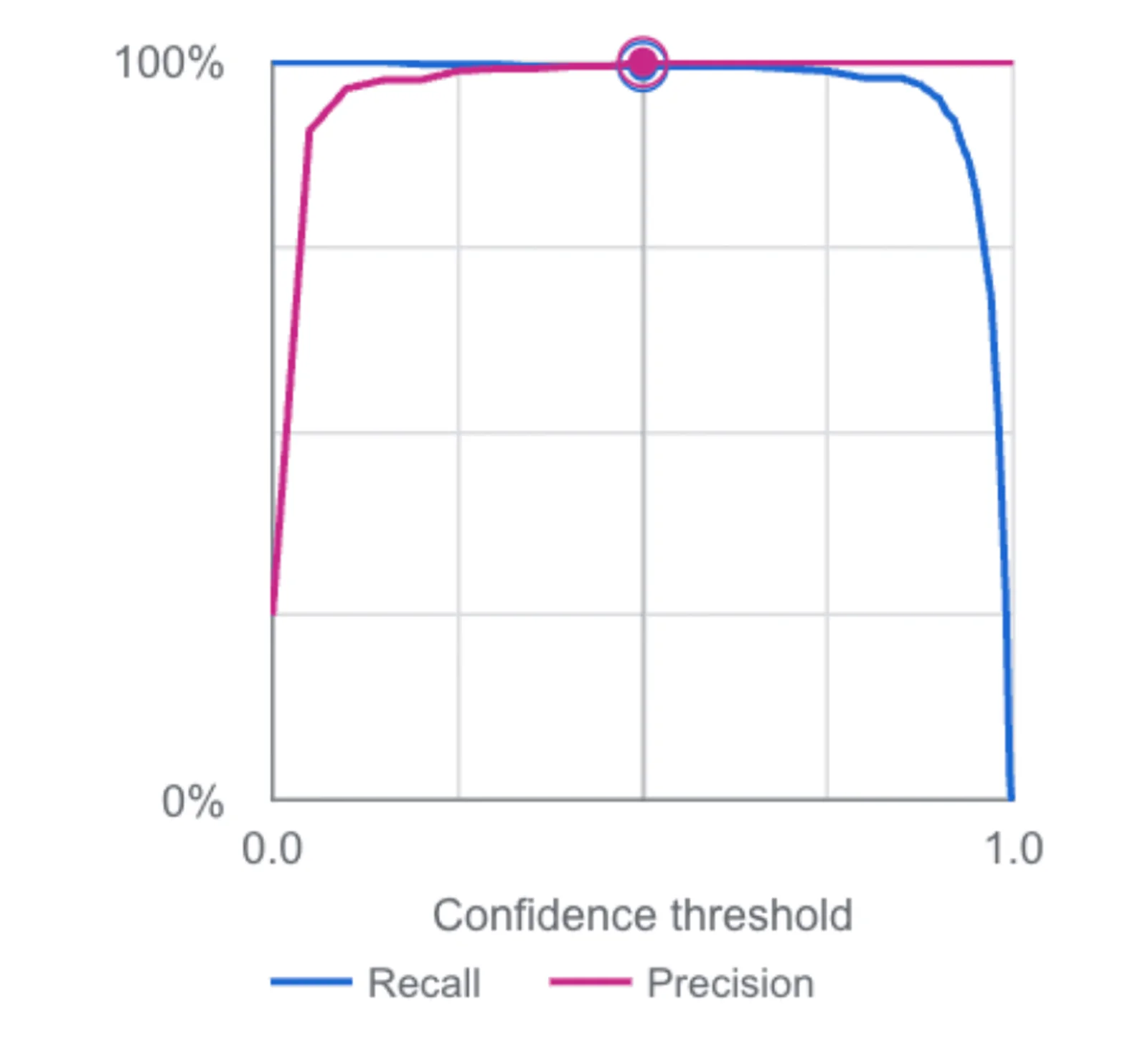
Effect of confidence threshold on model precision and recall Precision and recall are shown across increasing minimum-confidence requirements. The marked operating point represents the selected confidence threshold of 0.50, at which the model achieved 100% precision and 99.4% recall. Recall remained high across most of the evaluated threshold range but decreased as the required confidence approached its maximum.

The effect of prediction confidence was evaluated by comparing precision and recall across different minimum-confidence thresholds. Precision increased rapidly at lower thresholds and remained near 100% across most of the evaluated range. Recall also remained high through most of the threshold range but decreased sharply as the required confidence approached its maximum. At the selected confidence threshold of 0.50, the model achieved 100% precision and 99.4% recall (Figure 2).

Class-specific performance

**Figure 3:**
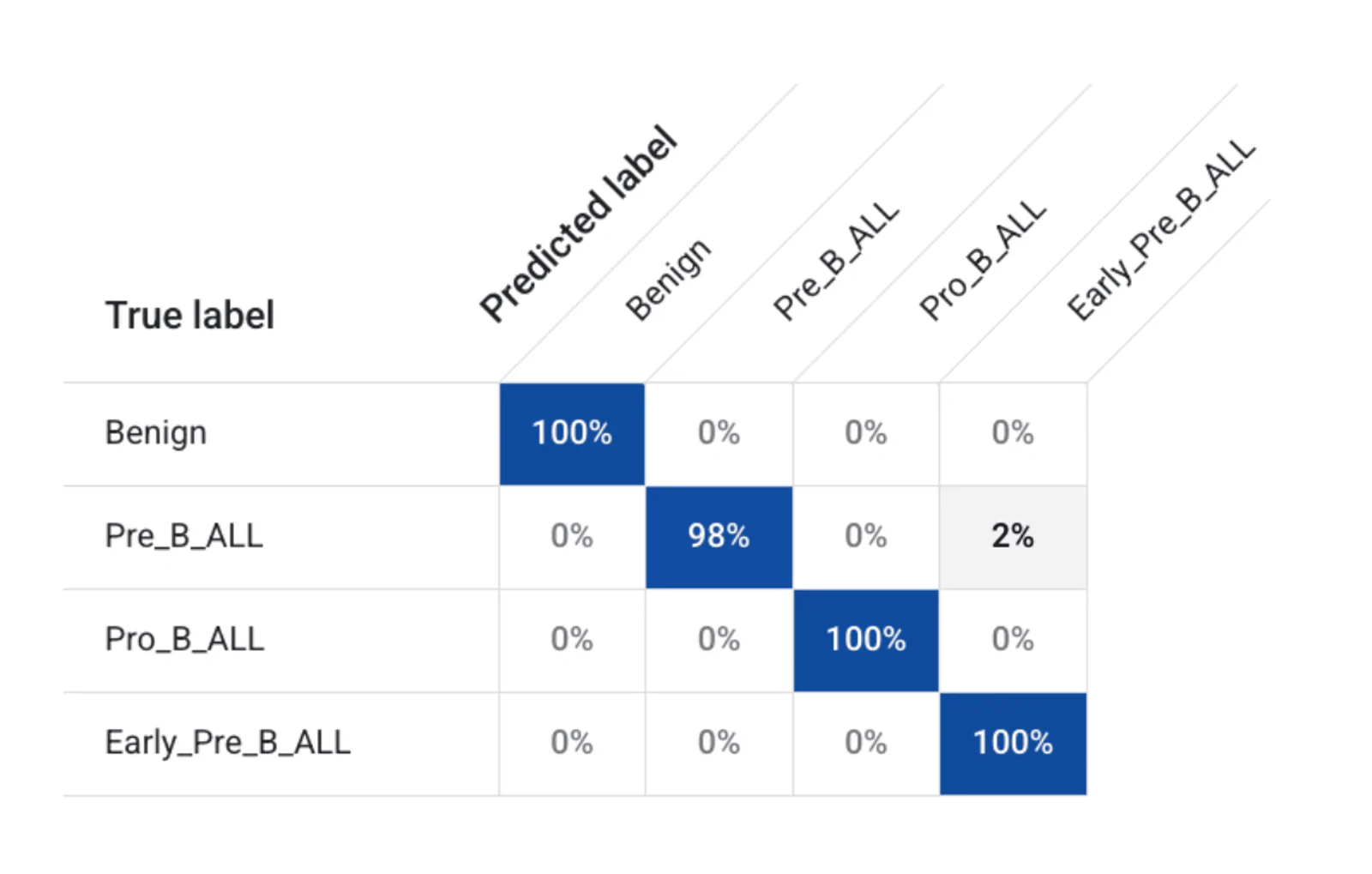
Confusion matrix for classification of benign hematogones and B-cell acute lymphoblastic leukemia (ALL) images Rows represent the true image categories, and columns represent the model-predicted categories. Diagonal values indicate correct classifications, while off-diagonal values indicate misclassifications. Pre-B ALL was correctly identified in 98% of cases, with 2% classified as early pre-B ALL. Benign hematogones, pro-B ALL, and early pre-B ALL each achieved 100% correct classification.

The multiclass confusion matrix demonstrated correct classification rates of 100% for benign hematogones, early pre-B ALL, and pro-B ALL images. Pre-B ALL images were correctly classified in 98% of cases, while the remaining 2% were classified as early pre-B ALL (Figure 3).

No benign hematogone images were classified as one of the leukemic categories, and no images from the three B-cell ALL categories were classified as benign. The observed errors were therefore limited to confusion between the pre-B and early pre-B ALL categories.

Representative model predictions

**Figure 4:**
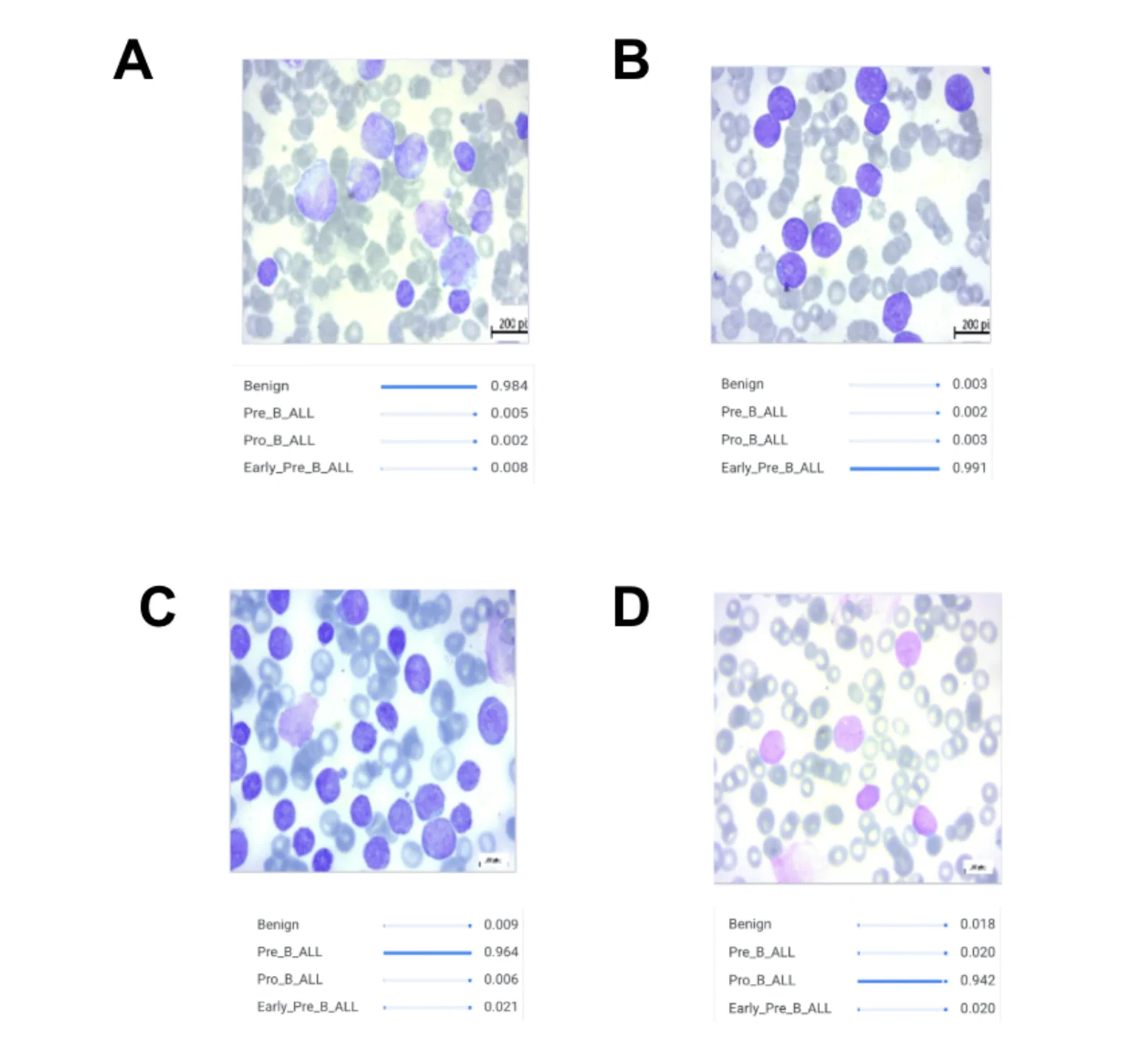
Representative peripheral blood smear images correctly classified by the artificial intelligence model Panel A shows a benign hematogone image, panel B shows an early pre-B acute lymphoblastic leukemia (ALL) image, panel C shows a pre-B ALL image, and panel D shows a pro-B ALL image. The model assigned the highest predicted probability to the correct category in each panel: 0.984 for benign hematogones, 0.991 for early pre-B ALL, 0.964 for pre-B ALL, and 0.942 for pro-B ALL. The accompanying values indicate the predicted probabilities assigned to all four classification categories. Representative cell images were obtained from the ALL Image Dataset hosted on Kaggle [20]. Classification probabilities were generated using the Google Cloud AutoML model developed in this study.

Representative images from each category were reviewed to illustrate the probability scores generated by the model (Figure 4).

The representative benign hematogone image received a predicted probability of 0.984 for the benign category. The model assigned predicted probabilities of 0.991, 0.964, and 0.942 to the representative early pre-B ALL, pre-B ALL, and pro-B ALL images, respectively. In each example, the correct category received the highest predicted probability, while the alternative categories received substantially lower scores.

## Discussion

This study evaluated whether a cloud-based AI model could distinguish benign hematogones from early pre-B, pre-B, and pro-B ALL in peripheral blood smear images. At the selected confidence threshold of 0.50, the model achieved an average precision of 1.00, a precision of 100%, and recall of 99.4%. The confusion matrix showed correct classification rates of 100% for benign hematogones, early pre-B ALL, and pro-B ALL and 98% for pre-B ALL. However, because the training and testing images were derived from the same publicly available dataset, patient-level separation and independence of image-acquisition conditions, including staining and microscopy characteristics, could not be confirmed. Accordingly, these findings should be interpreted as internal image-level performance and require validation using independently acquired, patient-level datasets. These findings demonstrate strong image-level performance within the internal testing dataset and indicate that the model differentiated closely related B-cell precursor categories with few errors.

The only observed misclassification occurred within the leukemic categories, where a small proportion of pre-B ALL images were classified as early pre-B ALL. Importantly, the model did not classify benign hematogones as leukemic or classify leukemic images as benign. The limited confusion between pre-B and early pre-B ALL may reflect overlap in their microscopic appearance because these categories are based primarily on immunophenotypic patterns of B-cell maturation rather than on a single distinctive morphologic feature. Differences in staining intensity, nuclear shape, cell orientation, image focus, or the amount of visible cytoplasm may also have influenced model predictions. Similar challenges have been described in prior image-based ALL classification studies, in which performance may be influenced by class imbalance, image quality, staining and illumination variability, preprocessing methods, and model architecture [16-18]. Explainability analyses have also identified morphologic features such as nuclear contour, chromatin appearance, and cytoplasmic characteristics as important contributors to model classification. Therefore, the observed confusion between pre-B and early pre-B ALL may reflect both genuine morphologic overlap between closely related B-cell maturation phenotypes and limitations in model feature extraction or training rather than a single identifiable cause. Because Google Cloud AutoML did not provide feature-level explainability outputs in the present study, the relative contribution of these factors could not be determined.

The findings are generally consistent with previous studies demonstrating that convolutional neural networks, transfer-learning systems, ensemble models, and explainable AI approaches can classify leukemia-related blood cell images with high internal performance [12-18]. Prior investigations have also shown that AI can distinguish benign hematogones from B-cell ALL lymphoblasts and differentiate among related B-lineage categories. The present study differs from many earlier investigations by using an automated cloud-based platform rather than a manually designed neural-network architecture. This approach allowed model development without requiring the investigators to select a specific network architecture or directly configure technical parameters such as learning rate, batch size, and optimization method.

Google Cloud AutoML offered several practical advantages, including automated feature extraction, model optimization, multiclass classification, and generation of category-specific probability scores. These functions may make medical image-classification research more accessible to investigators without advanced programming expertise or access to local high-performance computing resources. However, the automated design also reduces model transparency. The underlying neural-network architecture and training settings were not exposed to the investigators, limiting independent reproducibility and detailed examination of the model’s decision-making process. The representative images demonstrated that the correct category received the highest predicted probability in each selected example, but these images should be interpreted only as illustrations of model output rather than as additional evidence of performance beyond the complete testing dataset.

Several limitations should be considered when interpreting the results. First, the training, validation, and testing images were obtained from the same publicly available dataset. Images collected within a single source may share staining methods, microscope settings, camera characteristics, image backgrounds, and processing patterns that may not be present in other laboratories. The model may therefore have learned source-specific features, and its performance may decline when applied to images obtained from different institutions, microscopes, staining protocols, or patient populations.

Second, patient-level separation among the training, validation, and testing subsets could not be confirmed. If multiple images from the same patient were distributed across different subsets, similarities among those images could have contributed to the high reported performance. The findings should therefore be interpreted as an image-level internal evaluation rather than evidence of independent patient-level validation.

Third, the model classified individual microscopic images rather than complete clinical specimens. Diagnosis and classification of B-cell ALL require integration of morphology with clinical findings, flow cytometry, cytogenetic testing, and molecular analysis. The model was also evaluated only on the four categories included in the source dataset and was not tested on other leukemias, reactive conditions, technical artifacts, mature benign leukocytes, or additional benign precursor populations. Consequently, the model should not be considered an independent diagnostic system or a replacement for expert hematopathologic interpretation.

Future studies should evaluate the model using independent, multi-institutional datasets and ensure that all images from the same patient remain within a single data subset. External testing should include variation in staining methods, imaging equipment, magnification, image quality, and patient demographics. Comparisons with hematologists, hematopathologists, and laboratory specialists could help determine whether AI assistance improves classification accuracy, review time, or agreement among observers. Explainability techniques, such as activation maps or other region-based visualization methods, may also help determine whether the model is focusing on the target cell rather than on background features or image-acquisition artifacts.

Overall, the model demonstrated strong internal performance in distinguishing benign hematogones from three B-cell ALL categories. Misclassification was limited to a small proportion of pre-B ALL images classified as early pre-B ALL; however, these findings do not establish clinical generalizability. External, multi-institutional, and patient-level validation is required before clinical application. With further validation, cloud-based AI may serve as a supportive tool for microscopic blood cell analysis while remaining complementary to expert interpretation, flow cytometry, cytogenetic analysis, and molecular testing.

## Conclusions

This study demonstrated that a cloud-based AI model could accurately classify peripheral blood smear images as benign hematogones, early pre-B ALL, pre-B ALL, or pro-B ALL within an internal testing dataset. The model achieved strong overall performance, with misclassification limited to a small proportion of pre-B ALL images classified as early pre-B ALL.

These findings support the potential value of automated image classification as a supportive tool for microscopic blood cell analysis. However, the model was evaluated using images from a single publicly available dataset, and patient-level separation among the training, validation, and testing subsets could not be confirmed. External, multi-institutional, and patient-level validation is therefore required before clinical application. AI should remain complementary to expert hematopathologic interpretation, flow cytometry, cytogenetic analysis, and molecular testing rather than serve as an independent diagnostic system.
